# Running on the edge: hematological responses to a non-stop ultramarathon with a focus on nitric oxide–mediated red blood cell deformability

**DOI:** 10.3389/fphys.2025.1715513

**Published:** 2025-11-21

**Authors:** Marijke Grau, Jonas Bruns, Magnus Stücher, Lucas John, Moritz Munk, Michael Siebers, Christoph Siebers, Wilhelm Bloch, Daniel A. Bizjak

**Affiliations:** 1 Institute of Cardiovascular Research and Sports Medicine, Molecular and Cellular Sports Medicine, German Sport University Cologne, Cologne, Germany; 2 Department of Internal Medicine, Division of Sports and Rehabilitation Medicine, University Hospital Ulm, Ulm, Germany; 3 Institute of Forensic Psychiatry and Sex Research, Center for Translational Neuro- and Behavioral Sciences, University of Duisburg-Essen, Essen, Germany

**Keywords:** ultramarathon, hematological response, red blood cell deformability, nitric oxide, nitric oxide synthase, oxidative stress, endurance exercise

## Abstract

**Background/Objective:**

Ultramarathon competitions represent an extreme challenge for the human body, stressing physiological systems. However, little is known about their effects on the red blood cell (RBC) system, particularly on RBC deformability. Moreover, potential modulators of RBC deformability, such as oxidative stress and nitric oxide (NO) signaling, have not yet been systematically investigated in the context of ultramarathon running. The aim of the study was to assess alterations in these parameters following a 230 km non-stop ultramarathon, complemented by hematological parameters and markers of hematological stress to uncover mechanistic links.

**Methods:**

The investigation was conducted during the 2024 TorTour de Ruhr®. Twelve runners completed the race (3f/9m; mean age: 48.1 ± 6.9 years; 177.6 ± 8.0 cm; 71.3 ± 13.7 kg). Anthropometric data and venous blood sampling were collected pre- and post-race and analyzed using paired statistics.

**Results:**

Body weight as well as blood, plasma and RBC volumes remained unchanged. Post-race, mean corpuscular volume (MCV) and RBC distribution width (RDW) decreased, whereas mean corpuscular hemoglobin (MCH) and MCH concentration (MCHC) increased. These hematological shifts were associated with a leftward shift of the osmotic deformability curve, suggesting reduced RBC volume that could, over time, compromise cell integrity. Although acute hemolysis was not evident, significantly reduced haptoglobin levels indicate considerable cellular stress and raise the possibility of delayed hemolysis, warranting follow-up investigations. Importantly, RBC deformability improved post-race and was paralleled by elevated NO concentration, increased RBC-NO synthase activation, and enhanced S-nitrosylation of spectrins. These findings point toward activation of the RBC-NO pathway in response to mechanical stress, potentially supporting microcirculatory function. RBC free radicals decreased and total antioxidant capacity increased, suggesting a balanced redox response.

**Conclusion:**

Overall, the ultramarathon induced complex but compensatory adaptations in the RBC system, where NO-mediated improvements in deformability may counteract stress-induced risks to cell integrity. Thus, despite no acute impairment of RBC function, adequate hydration, antioxidant-rich nutrition, and sufficient recovery may be emphasized to safeguard long-term RBC health in extreme endurance exercise.

## 1 Introduction

Ultramarathon runs, defined as distances exceeding 42.195 km, are becoming increasingly popular with several hundred ultramarathon events held annually across Europe (German Ultramarathon Association). Regular endurance exercise is known to improve cardiorespiratory fitness, oxygen transport capacity, and oxygen diffusion to skeletal muscles, while participation in such extreme events places substantial physiological demands on the human body, challenging cardiovascular, hematological, and musculoskeletal systems (Kokkinos et al., 2010; Ruegsegger and Booth, 2018; Bizjak et al., 2022; Nikolaidis et al., 2018; Hoppel et al., 2019).

In addition, endurance training can enhance hemorheological properties, including Red Blood Cell (RBC) deformability, a key determinant of microcirculatory blood flow (Bizjak et al., 2020). RBC deformability is influenced by the viscoelastic properties of the cell membrane, cytosolic viscosity determined via intracellular hemoglobin concentration, and cell geometry (Baskurt and Meiselman, 2003; Kim et al., 2012). Endurance athletes generally exhibit higher deformability, partly due to a greater proportion of young, flexible RBC (Bizjak et al., 2020; Tomschi et al., 2018; Smith, 1995). RBC deformability is also modulated by nitric oxide (NO), which is enzymatically produced by RBC-NO synthase (RBC-NOS) (Bor-Kucukatay et al., 2003; Kobayashi et al., 2022; Grau et al., 2013; Kleinbongard et al., 2006). RBC-NOS activation and subsequent NO production increase in response to shear stress (Kuck et al., 2019; Horobin et al., 2021), suggesting that the enhancement of deformability during exercise may be at least partly NO-dependent. NO-mediated regulation of RBC deformability is crucial for tissue oxygen delivery, highlighting its importance in extreme endurance events.

Despite these adaptations, extreme physical stress such as ultramarathon training and competition may overwhelm physiological systems, potentially leading to oxidative stress and hemolysis (Arakawa et al., 2016; Hattori et al., 2009). Elevated reactive oxygen species (ROS) have been reported during multi-day ultramarathons, which may compromise RBC integrity and function (Arakawa et al., 2016; Hattori et al., 2009; Kuck et al., 2019; Pandey and Rizvi, 2011; Carin et al., 2025). Data on hematological responses to ultramarathons are inconsistent: some studies report increased markers of hemolysis, such as bilirubin and reduced haptoglobin (Arakawa et al., 2016; Liu et al., 2018), whereas others show only modest reductions in haptoglobin with stable hematocrit (hct), hemoglobin concentration (hb), and RBC count post-race (Robach et al., 2014; Robert et al., 2020; Lippi et al., 2012). In certain cases, RBC count, hb and mean corpuscular volume (MCV) even increased after an ultramarathon (Dickson et al., 1982).

To date, only a few studies have directly examined RBC deformability in ultramarathon participants. Robert et al. reported either unchanged or decreased deformability depending on race length (Robert et al., 2020), while Liu et al. observed alterations in RBC viscoelastic properties with divergent trends among participants (Liu et al., 2018). In contrast, Carin et al. found increased RBC deformability irrespective of running distance. The mechanisms underlying these discrepancies remain unclear, highlighting the need for further investigation into the factors that modulate RBC mechanical properties during prolonged endurance exercise.

Overall, hematological responses to ultramarathons appear highly variably, likely influenced by race duration, conditions, and participant characteristics. Moreover, the impact of ultramarathon running on RBC deformability and its NO-mediated regulation remains poorly understood. This study therefore aimed to investigate the effects of a non-stop ultramarathon on RBC deformability and NO parameters, as well as hematological and oxidative stress markers, in participants of the TorTour de Ruhr®. The aim was to elucidate whether ultramarathon running may pose a potential risk to RBC integrity and function.

## 2 Materials and methods

### 2.1 Participation requirements, race conditions, and information regarding sample collection

Participation in the TorTour de Ruhr® required a personal invitation from the organizer and successful completion of previous ultramarathons. On average, the runners had completed more than 60 ultramarathons. Participants were required to provide a medical certificate confirming their physical resilience. The following exclusion criteria were defined to avoid interference with the study parameters: nicotine consumption; blood clotting disorders or intake of blood thinning medications; acute or chronic vascular disorders; cardiovascular, metabolic or autoimmune diseases.

All participants provided written informed consent. The protocols described here are in alignment with the Declaration of Helsinki and were approved by the Ethics Committee of the German Sports University Cologne (012/2024).

Initially, fifteen ultramarathon runners were included in this study of which three participants dropped out during the race. Thus, data from a total of twelve participants (3f/9 m) were included in the final analysis. Anthropometric measures were carried out pre- and post-race using a bio-impedance scale (InBody 770, InBody Europe B.V., Eschborn, Germany) (Table 1). Fluid intake was documented by the participants themselves or their personal crew.

**TABLE 1:** Anthropometric data and blood volume of study participants.

Parameter	Pre-race	Post-race	p-value
Height [cm]	177.6 (8.0)		
Body mass [kg]	71.2 (13.7)	72.5 (15.5)	0.382
Blood volume [L]	4.86 (0.8)	4.92 (0.9)	0.222
Plasma volume [L]	2.86 (0.4)	2.95 (0.5)	0.155
RBC volume [L]	2.01 (0.4)	1.97 (0.4)	0.229

Data are Mean (SD) of n = 12.

Venous blood was drawn from a prominent antecubital vein into EDTA vacutainers (BD, Heidelberg, Germany) both pre- and post-race. Pre-race sampling took place in Winterberg, Germany, on 17 May 2024, between 4 and 8 p.m. The 230 km race started 18 May 2024, at 8 a.m. Post-race blood sampling was performed in Duisburg, Germany, within 10 min of crossing the finish line. Mean completion time for the 230 km ultramarathon was 32 h 52 min 34 s. Blood samples were immediately processed, with aliquots either stored at −20 °C or −80 °C, respectively, for later analysis or measured on-site as indicated below. Weather conditions at the start were 9.2 °C with 85% relative humidity. At the finish, temperatures ranged from a daily minimum of 12.2 °C to a maximum of 17.8 °C, with an average of around 15 °C. In the afternoon, values peaked near 19 °C before dropping to about 18 °C in the evening, accompanied by a light westerly breeze, intermittent heavy rain, and humidity levels of approximately 64%–66%.

### 2.2 Complete blood count, plasma haptoglobin, lactate and RBC electrolyte measurements

A complete blood count was performed at a medical laboratory (Dr. Wisplinghoff, Cologne, Germany). Only the relevant RBC-related parameters are reported in this manuscript.

For blood lactate concentration measurements, 20 µL of whole blood was mixed with hemolyzing solution (EKF Diagnostic Sales, Magdeburg, Germany) and lactate concentration was measured using the EKF Biosen C-Line Analyser (EKF Diagnostics GmbH, Barleben, Germany).

One aliquot of whole blood was separated by centrifugation (3,700 × g for 10 min at 4 °C), and RBC were diluted (1:10) with phosphate buffered saline (PBS; 0.1 mol; pH 7.4). Of these samples, RBC sodium (Na^+^), and potassium (K^+^) concentrations were measured using flame photometry (EFOX 5053; Eppendorf AG, Hamburg, Germany). Plasma samples were used to measure haptoglobin concentration using the Human Haptoglobin ELISA Kit according to the manufacturers’ description (Abcam, Cambridge, United Kingdom).

### 2.3 RBC deformability and RBC Osmoscan

Measurements of RBC deformability by ektacytometry and under an osmotic gradient (=Osmoscan) were directly performed using the laser-assisted optical rotational red cell analyzer (Lorrca MaxSis, RR Mechatronics, Zwaag, Netherlands) as previously described (Hardeman et al., 2001). Whole blood was separated by centrifugation (3,700 × g, 10 min) and 100 × 10^6^ RBC were mixed with 5 mL of an isotonic viscous medium (0.14 mM Polyvinylpyrrolidone [PVP], 29.7 cP at 37 °C, RR Mechatronics, Zwaag, Netherlands). This solution was placed in a Couette system and nine consecutive shear stresses between 0.3 and 30 Pa were applied to the samples. The Lorrca system (software version 5.04) converted the diffraction pattern of a laser beam passing through the samples into an Elongation Index (EI) which was then used by the Lorrca system to calculate the following parameters: maximum deformability at infinite shear stress (EImax) and shear stress at one-half of EImax (SS1/2).

The Osmoscan test reflects the condition of the cells across different osmotic values. It delivers insights into their deformability and membrane rigidity, based on both the shape of the curve and its position along the osmolality axis. For osmoscan measurements, 1000 × 10^6^ RBC were mixed with 5 mL of PVP (see above). The Osmoscan was automatically measured by the Lorrca MaxSis. Results included Omin, reflecting the osmolality at minimum RBC deformability, below which RBC would lyse with further decreases in osmolality. EImax represents the maximum deformability at isotonicity, and Ohyper reflects the hyperosmotic osmolality corresponding to 50% EImax.

### 2.4 RBC nitrite/RSNO/iron-nitrosylheme

RBC nitrite/RSNO/iron-nitrosylheme concentration was measured using an ozone-based chemiluminescence NO detector (CLD 88e, EcoPhysics AG, Duernten, Switzerland) as previously described (Pelletier et al., 2006; Grau et al., 2007). Briefly, whole blood was centrifuged (see above) and RBC were mixed with a nitrite preservation solution and stored at −80 °C until needed. Prior to measurement, samples were thawed on ice, mixed with ice-cold methanol (VWR International GmbH, Darmstadt, Germany) in a 1:2 ratio and centrifuged at 3,600 × g, 4 °C for 20 min. Supernatant was injected into an acidified tri-iodide solution which reduces nitrite/RSNO/iron-nitrosylheme to NO gas which was then analyzed via its gas-phase chemiluminescent reaction with ozone. Data analysis was performed using the Chart FIA software (eDAQ Chart v.5.5.11; Ecophysics, Switzerland) to integrate the area under the curve. A standard curve was used to calculate nitrite/RSNO/iron-nitrosylheme within the samples after correction of the concentration for nitrite/RSNO/iron-nitrosylheme levels of the used methanol and preservation solution. Each sample was measured in triplicate.

### 2.5 Immunostaining of RBC-NOS serine 1177 residue

The immunostaining protocol has been published in detail elsewhere (Grau and Kuck, 2023). Briefly, whole blood was incubated with 4% formaldehyde solution and fixed RBC were then dispersed on a slide. The staining procedure included the following major steps: (1) opening of cell membrane, (2) blocking peroxidase activation, (3) application of primary antibody (Rabbit anti Human NOS III Serine 1177; 1:500; Merck, Darmstadt, Germany), (4) application of secondary antibody (goat anti-rabbit antibody; biotinylated; 1:150; Dako, Glostrup, Denmark), (5) application of avidin-coupled horseradish peroxidase (1:400; Sigma/Merck, Darmstadt, Germany) and (6) development of staining using 3,3-diaminobenzidine-tetrahydrochloride solution (Sigma-Aldrich, St. Louis, United States). Samples were then dehydrated using increasing alcohol solutions. Mounting medium Entellan® (Merck, Darmstadt, Germany) was applied and samples were covered. Images of the stained RBC were taken using an Axiophot 1 microscope (Zeiss, Oberkochen, Germany) coupled to a camera (Progres Gryphax Prokyon; Jenoptik Optical Systems GmbH, Jena, Germany) with a 400-fold magnification. Staining was analyzed with software ImageJ 1.52a (National Institutes of Health, Bethesda, United States). For staining intensity analysis, mean gray values were measured, and total immunostaining intensity was calculated (Grau and Kuck, 2023).

### 2.6 S-nitrosylation of RBC cytoskeletal α- and β-spectin

The S-Nitrosylated Protein Detection Assay (Cayman Chemical, Ann Arbor, United States) was performed following established protocols (Grau et al., 2013). During this procedure, RBC were lysed and free thiol (SH) groups were blocked to avoid unspecific reactions during subsequent labeling steps. S-nitrosothiol bonds were then selectively cleaved, releasing SH groups, which were subsequently tagged with a biotin reagent to enable specific detection. Then, protein concentrations were determined with the DC Protein Assay Kit (Bio-Rad, Munich, Germany). Equal amounts of protein (60 µg) were loaded alongside the Precision Plus Protein™ Standard (Bio-Rad) onto 4%–12% Criterion™ XT Bis-Tris gels (Bio-Rad) and electrophoresed for 90 min at a constant current of 90 mA in 1× XT MOPS running buffer (Bio-Rad). Proteins were then transferred onto membranes (Trans-Blot Turbo Transfer Pack; Bio-Rad) using 25 V for 30 min. Membranes were blocked with 2% BSA in 1× TBST prior to labeling with horseradish peroxidase (HRP; 1:1000 dilution in 1× TBST). Signal detection was carried out by enhanced chemiluminescence (ECL) using the ChemiDOC MP imaging system (Bio-Rad). Band intensities were quantified with ImageJ software by measuring the integrated density.

### 2.7 Parameters of oxidative stress: free ROS/RNS levels and total antioxidant capacity

RBC free radical content was measured in 4 × 10^6^ RBC using the OxiSelect *In Vitro* ROS/RNSAssay Kit (Cell Biolabs Inc., San Diego, United States), as previously described (Grau et al., 2021), and resulting fluorescence was read with a fluorescence plate reader (Fluoroskan Ascent Microplate Fluorometer; Thermo Fisher Scientific, Waltham, United States) at 480 nm excitation and 530 nm emission.

Total antioxidant capacity (TAC) was measured in 1 × 10^7^ RBC using a respective Assay Kit (Abcam, Cambridge, United Kingdom). Absorbance was measured at 570 nm using a Multiskan FC Photometer (Thermo Fisher Scientific, Waltham, United States). Antioxidant capacity was expressed as Trolox equivalent capacity, following the principle that the sample´s antioxidant capacity is compared to that of Trolox (Grau et al., 2021).

### 2.8 Statistics

The software GraphPad Prism (GraphPad Prism 9.5.1; San Diego, CA, United States) was used for statistical analysis and preparation of presented figures. Data were tested for Gaussian distribution using the Shapiro-Wilk test (Mishra et al., 2019). Either a two-tailed paired t-test (data passed normality test) or a Wilcoxon matched-paired signed rank test (data did not pass normality test) was used to compare values measured pre- and post-race. Pearson correlation was performed to test the relation between mean corpuscular hemoglobin concentration (MCHC) and Ohyper. Data are presented as mean ± standard deviation as well as changes in individual values from pre- to post. Statistical significance was established at p ≤ 0.05.

## 3 Results

### 3.1 Anthropometric measures, blood volume and fluid intake

Anthropometric measures are presented in Table 1. Values of body mass did not significantly differ between pre- and post-race. Participants’ height, body mass, hematocrit and sex were used to calculate blood, plasma and RBC volume using the Nadler equation as previously published (Hoffman et al., 2018), and respective values did not differ post-race. On average, the participants consumed 12.55 ± 9.1 L of fluids during the race (min: 4.22 L; max: 31.85 L).

### 3.2 RBC indices, plasma haptoglobin concentration, lactate levels and RBC electrolyte concentration

RBC count, hemoglobin concentration (hb) and hematocrit (hct) were unaffected by the race. Post-race, mean corpuscular volume (MCV), red blood cell distribution width (RDW) and plasma haptoglobin concentration decreased significantly, whereas mean corpuscular hemoglobin (MCH) and MCHC increased. Lactate concentration also increased post-race. RBC sodium levels remained comparable before and after the race, while potassium levels decreased significantly (see Table 2 for all values).

**TABLE 2:** Hematological and biochemical parameters related to RBC of study participants.

Parameter	Pre	Post	p-value
RBC [*10^6^/µL]	4.59 (0.3)	4.63 (0.3)	0.322
Hb [g/dL]	13.74 (0.9)	13.83 (0.7)	0.361
Hct [%]	41.50 (2.6)	40.50 (2.0)	0.099
MCV [fL]	90.17 (4.5)	88.08 (3.7)	**<0.001**
MCH [pg]	30.00 (1.9)	30.25 (2.0)	**0.040**
MCHC [g/dL]	33.25 (1.1)	34.17 (0.9)	**0.004**
RDW [%]	12.94 (0.9)	12.69 (0.9)	**0.003**
Plasma haptoglobin [mg/dL]	128.17 (164.8)	66.82 (82.8)	**0.042**
Lactate [mmol/L]	1.46 (0.4)	2.53 (1.2)	**0.006**
RBC sodium [mmol/L]	59.91 (20.9)	52.47 (15.9)	0.146
RBC potassium [mmol/L]	9.12 (1.7)	7.41 (1.1)	**0.002**

Data are Mean (SD) of n = 12. Significant differences between pre-and post-measurements (p < 0.05) are shown in bold.

RBC, red blood cells; Hb, hemoglobin concentration; Hct, hematocrit; MCV, mean corpuscular volume; MCH, mean corpuscular hemoglobin; MCHC, mean corpuscular hemoglobin concentration; RDW, RBC, distribution width.

### 3.3 Red blood cell deformability and Osmoscan

**FIGURE 1:**
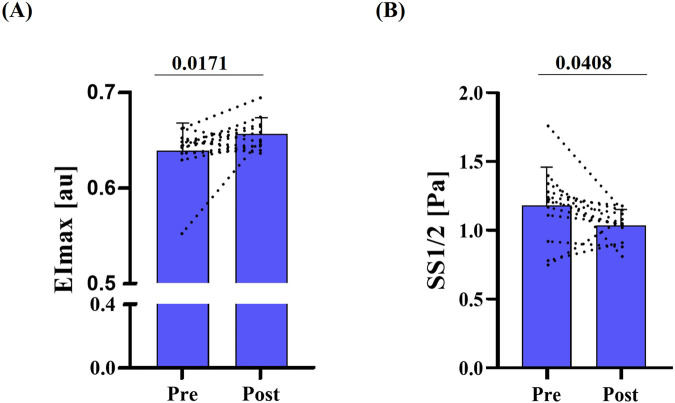
RBC deformability pre- and post-race. **(A)** Maximum deformability (EImax) was significantly higher post-race (p = 0.0171). **(B)** Shear rate required for half EImax (SS1/2; Pa) was lower post-race; also indicating higher deformability behavior of RBC (p = 0.0408). Data are presented as mean ± SD (n = 12).

**FIGURE 2:**
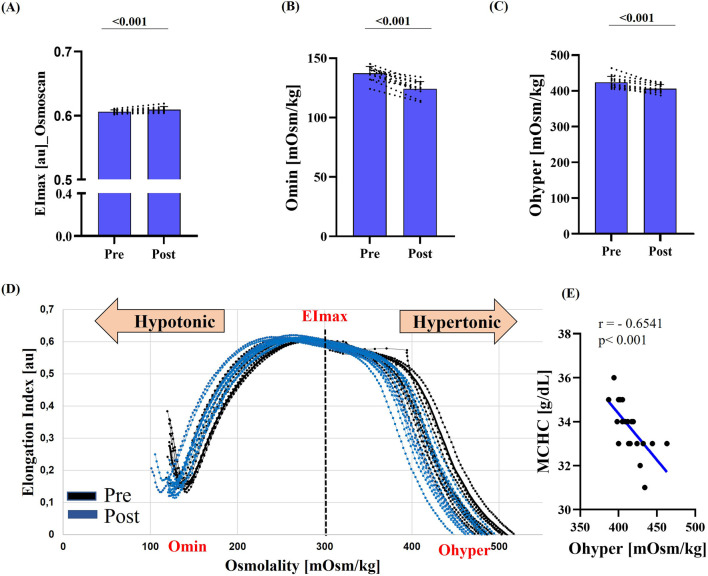
RBC deformability under osmotic gradient (Osmoscan) pre- and post-race. **(A)** Maximum deformability (EImax) was significantly increased post-race (p < 0.001). **(B)** Omin and **(C)** Ohyper were significantly lower post-race (both p < 0.001). **(D)** Representative Osmoscan curves pre-race (black) and post-race (blue) illustrate a marked leftward shift. **(E)** Correlation analysis showed a strong negative relationship between MCHC and Ohyper (r = −0.6541; p < 0.001). Data are presented as mean ± SD (n = 12).

RBC deformability, assessed by standard ektacytometry, increased post-race, as indicated by a significant rise in EImax (p = 0.017) and a significant reduction in SS1/2 (p = 0.041) (Figures 1A,B). Similarly, EImax measured during Osmoscan was significantly higher post-race (p < 0.001) (Figure 2A). Omin and Ohyper decreased significantly after the race (p < 0.001) (Figures 2B,C), which was also reflected by a leftward shift of the corresponding curve (Figure 2D).

Correlation analysis revealed a strong inverse relationship between MCHC and Ohyper (r = −0.6541; p < 0.001).

### 3.4 RBC-NOS activation and NO-dependent spectrin modifications

**FIGURE 3:**
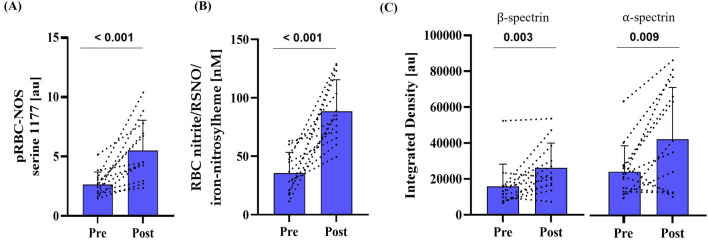
Activation of RBC-NOS and subsequent NO-mediated post-translational modification of cytoskeletal spectrin pre- and post-race. **(A)** Phosphorylation of RBC-NOS at serine 1177 residue (p < 0.001), **(B)** RBC nitrite/RSNO/iron-nitrosylheme concentration (p < 0.001) and **(C)** S-nitrosylation of cytoskeletal spectrins (α-spectrin: p = 0.009; β-spectrin: p = 0.003) were increased compared to pre-race conditions. Data are presented as mean ± SD (n = 12).

Staining intensity of phosphorylated RBC-NOS at serine 1177 residue, and thus activation of the enzyme, significantly increased post-race (p < 0.001) (Figure 3A). Levels of RBC nitrite/RSNO/iron-nitrosylheme were significantly higher post-race (p < 0.001) (Figure 3B). S-nitrosylation of α- and β-spectrin, a post-translational modification, both increased significantly after the race (α-spectrin: p < 0.001; β-spectrin: p = 0.005) (Figure 3C).

### 3.5 Markers of oxidative stress: free ROS/RNS and total antioxidant capacity

**FIGURE 4:**
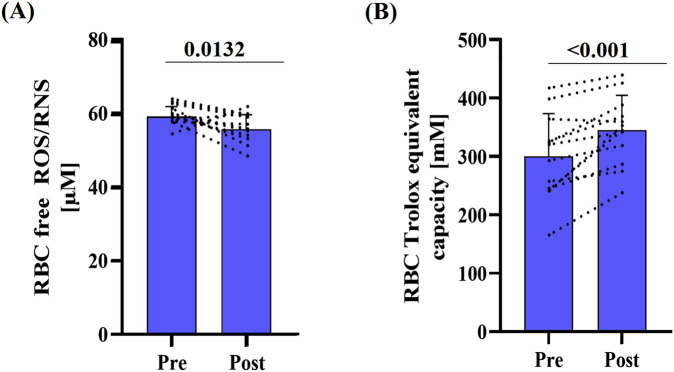
Marker of oxidative stress pre- and post-race. **(A)** RBC free ROS/RNS decreased post-race (p < 0.0132), while **(B)** mean total antioxidant capacity, expressed as Trolox equivalent capacity, significantly increased (p < 0.001). Data are presented as mean ± SD (n = 12).

Free RBC ROS/RNS levels decreased significantly after the race (p = 0.0132) (Figure 4A). In parallel, RBC total antioxidant capacity, as indicated by RBC Trolox equivalent capacity, was elevated post-race (p < 0.001) (Figure 4B).

## 4 Discussion

The growing popularity of ultramarathon running illustrates that extreme physical exertion is becoming increasingly common. However, the physiological strain such events impose on body systems such as the RBC system remains largely unexplored, and the underlying mechanisms are still poorly understood.

This study revealed a substantial increase in RBC deformability, which was accompanied by activation of the RBC-NOS-NO pathway and enhanced S-nitrosylation of cytoskeletal spectrins. RBC oxidative status was not adversely affected by the ultramarathon. Furthermore, Ohyper was reduced, and this reduction was associated with increases in MCHC. Together with the decreases in MCV, RDW and Omin, these findings may indicate a reduced cell hydration status, which could potentially affect RBC characteristics to a later stage. Reductions in haptoglobin concentration suggested RBC lysis and thus increased mechanical stress on the cells. However, total RBC count remained unaltered, implying that the deleterious impact of mechanical load on RBC integrity was relatively minor. In this context, although blood lactate concentrations were significantly higher post-race, they remained within a moderate range (1.3 and 5.99 mmol/L), suggesting that metabolism was, for all but one runner, below the aerobic/anaerobic threshold at the end of the race. This is likely related to the relatively slow running speed of approximately 7 km/h and the 7–9 mandatory stops at checkpoints.

### 4.1 Alterations in blood profile

Body weight was not affected by the race, which contrasts with comparable studies reporting decreased body weight after long endurance runs (Costa et al., 2013; Knechtle and Nikolaidis, 2018; Kao et al., 2008). Additionally, calculations of blood-, plasma- and RBC-volumes remained unchanged, and hematocrit was not significantly altered, suggesting no signs of hemoconcentration in the runners. Several recommendations advise fluid intake of 400–800 mL/h, depending on individual runners and weather conditions (Armstrong, 2021). For a race duration of 26–36 h as like in this setting, this would correspond to 10.4–13.2 L for a fluid intake of 400 mL/h. According to the provided self-reports, participants of this ultramarathon consumed a mean of 392 mL/h (range 124–884 mL/h) in fluids, excluding high-water-content foods. This level of fluid intake, combined with the low running velocity, the efficient sweating associated with the good training status, and the relatively cool to moderate temperatures during the run–which likely resulted in lower fluid loss through sweating compared to hot conditions–appears to have been sufficient to maintain an adequate fluid balance.

Consistent with previous studies (Lippi et al., 2012; Rama et al., 2016; Fallon et al., 1999), the present results demonstrated a significant decrease in MCV and RDW, accompanied by an increase in MCHC. Simultaneously, the Osmoscan curve, which reflects RBC deformability under an osmotic gradient, exhibited a leftward shift on both curve tails (Omin and Ohyper), suggesting a certain degree of cell dehydration (Krishnevskaya et al., 2023). This shift is reported to be influenced by changes in MCV and MCHC, and correlation analyses indeed revealed a strong negative association between MCHC and Ohyper. Variations in electrolyte levels were reported to affect RBC volume. In this study, measured RBC sodium levels were unchanged after the race, whereas RBC potassium significantly decreased post-race, consistent with previous reports (Lijnen et al., 1989). These reductions may reflect potassium loss from the plasma, e.g., caused by mechanical load (Vøllestad et al., 1994), supporting the hypothesis that RBC can act as a potassium reservoir (Lijnen et al., 1989). The resulting decrease in intracellular osmotic activity likely leads to water efflux into the extracellular space, causing functional cell shrinkage, which may explain the observed reduction in MCV. The reduced RDW observed in the present study may also be related to the decreased MCV values, as RDW is calculated as the standard deviation of (MCV/MCV) × 100 and reflects the degree of heterogeneity in RBC volumes. Therefore, although a loss of body water may be negligible, an intracellular fluid shift can be assumed, which could induce lasting alterations in the RBC without optimal recovery.

RBC markers, including RBC count, hemoglobin concentration, and haptoglobin levels, were measured to assess hemolysis, which has been reported to occur during prolonged training sessions and endurance events. This phenomenon, known as foot-strike hemolysis, refers to RBC damage caused by mechanical stress within the capillaries as a result of the repeated, forceful impact of the feet to the ground, leading to intravascular hemolysis (Lippi et al., 2012; Telford et al., 2003). Plasma haptoglobin levels decreased after the run, indicating some degree of RBC damage. In contrast, RBC count and hemoglobin concentration remained unchanged, suggesting that mechanically induced hemolysis might be negligible immediately post-race, as reported by several studies (Robert et al., 2020; Lippi et al., 2012). This could be attributed to improvements in footwear technology and cushioning (Dressendorfer et al., 1992; Falsetti et al., 1983), as well as the relatively low running speed. Since data were collected only immediately post-race, RBC might still sustain lasting damage or undergo delayed lysis. Future follow-up studies are therefore needed to determine whether this type of stress eventually affects RBC counts through subsequent cell lysis.

### 4.2 RBC deformability: implications of NO production and oxidative stress

RBC deformability is a unique cellular property that enables RBC to traverse the microcirculation for oxygen delivery (Huisjes et al., 2018). This property is determined by the surface-to-volume ratio, internal viscosity and membrane elasticity (Clark et al., 1983; Baskurt and Meiselman, 2003). RBC deformability has been reported to be influenced by mechanical stress, including endurance exercise, with the intensity and duration of the activity determining the extent of this effect. High-intensity exercise is generally associated with a reduction in deformability, whereas low-intensity exercise appears to have no or only minimal effects. In contrast, moderate to vigorous exercise has been shown to enhance RBC deformability (Suhr et al., 2009; Koliamitra et al., 2017; Mairbäurl, 2013). However, the overall response is not solely determined by exercise intensity but also by its duration. Short bouts of exercise, regardless of intensity, tend to induce little or no change in RBC deformability, while prolonged endurance activity can lead to more pronounced alterations (Bizjak et al., 2020; Koliamitra et al., 2017; Smith, 1995; Simmonds et al., 2013; Nemkov et al., 2021). As an extreme form of prolonged endurance exercise, ultramarathon running provides a unique model to study such effects under maximal physiological strain. A study by Liu et al. (2018) suggests–although the data are not conclusive–that ultramarathon participation may reduce the viscoelastic properties of RBC, whereas data of Robert et al. (2020) found no changes in RBC deformability after a 40 km run. Conversely, deformability was reduced following a 171 km run with an elevation gain of 10,000 m (Robert et al., 2020). The data from the present study align with recent findings by Carin et al. (2025), demonstrating increases in RBC deformability measured by both standard ektacytometry and osmotic gradient-based deformability assessments.

RBC deformability is influenced by RBC-derived NO (Grau et al., 2013). Within RBC, NO is enzymatically produced by RBC-NOS (Kleinbongard et al., 2006). RBC-NOS activation, indicated by phosphorylation of the serine 1177 residue, increased significantly post-race and was paralleled by increased RBC-nitrite/RSNO/iron-nitrosylheme concentrations as well as S-nitrosylation of the α- and β-spectrins. These findings suggest that, consistent with earlier reports (Suhr et al., 2012; Grau et al., 2013), the shear stress experienced by the RBC during the prolonged run activated the RBC-NOS/NO signaling pathway, thereby positively influencing RBC deformability during the race. Functionally, this NO-mediated modulation of the membrane/cytoskeleton appears to act as an acute, compensatory response that preserves microcirculatory passage and oxygen delivery despite stressors to RBC volume.

Not only mechanical stress but also free radicals have been reported to influence RBC-NOS activation and RBC deformability (Kuck et al., 2019; McNamee et al., 2018). Oxidative stress can induce RBC stiffening, which increases susceptibility to shear-related damage (McNamee et al., 2018) and may compromise oxygen delivery (Mohanty et al., 2014). In the context of ultralong endurance exercise, such limitations in oxygen transport could impair aerobic energy production, accelerate fatigue and ultimately reduce performance capacity. Studies by Spanidis et al. and Carin et al. reported increased levels of stress markers such as static oxidation-reduction potential marker, hydrogen peroxide and isoprostane levels following ultramarathon races (Spanidis et al., 2017; Carin et al., 2025). Interestingly, the magnitude of this stress response appears to depend on race distance, with shorter ultramarathons eliciting a more pronounced increase in hydrogen peroxide and isoprostane compared to longer events (Carin et al., 2025). In the present study, post-race measurements revealed a decrease in free RBC ROS and RNS levels, which implies that oxidative stress within RBC is unlikely to be elevated during this prolonged endurance exercise. These findings do not fully align to the above cited studies which describe increased oxidative stress in RBC under similar conditions. To obtain a more comprehensive understanding of exercise-induced oxidative stress, future studies should include additional markers beyond free ROS/RNS. Also, the reductions in free RBC ROS/RNS presented here do not preclude the presence of systemic oxidative stress, which may affect other tissues or circulating plasma components and could still influence overall physiological strain and recovery as previously reported (Bizjak et al., 2022). These findings may suggest a compartmentalized redox response, in which RBC may be protected or adaptively regulated (Spinelli et al., 2025) to maintain deformability and oxygen delivery despite systemic stress.

Additionally, in response to exercise, glucagon, catecholamines, and vasopressin stimulate the release of glutathione into the plasma, which can be taken up by the heart, muscles and also RBC, potentially to counterbalance the increased production of free radicals (Sies and Graf, 1985; Maxwell et al., 1993; Ji et al., 1998). Measurement of TAC thus provides valuable information on the systemic antioxidative capacity and can contribute to the assessment of oxidative stress. While Spanidis et al. observed no changes in TAC 24 h after an ultramarathon (Spanidis et al., 2017), others reported increases in TAC following marathon and ultramarathon events, respectively (Stagos et al., 2015; Jastrzębski et al., 2016; Skenderi et al., 2008). The data of the present study are in line with these reports, suggesting that the high antioxidative capacity within RBC counteracts potential oxidative stress. This mechanism may also play a role in preserving RBC-NOS dependent NO production and further RBC deformability.

In summary, the post-race increase in RBC deformability is likely attributable to the beneficial effects of prolonged, low-speed running, which appears to enhance deformability in a RBC-NOS/NO-dependent manner, thereby facilitating microcirculatory passage and improving tissue oxygen delivery, muscle perfusion, and recovery following prolonged endurance exercise. This adaptation seems to be supported by the high antioxidative capacity within RBC, which mitigates oxidative/nitrosative stress and helps preserve RBC function under systemic stress conditions.

### 4.3 Limitations

This study has some limitations that should be considered. Only twelve participants were included in the sample analysis. However, sample sizes in comparable studies are of similar magnitude (Jastrzębski et al., 2016; Robert et al., 2020; Fallon et al., 1999; Lavoué et al., 2020; Spanidis et al., 2017; Stagos et al., 2015), reflecting the challenging nature of this endurance sport.

Women were underrepresented in the study cohort, preventing sex-specific analysis of the outcomes. It is well established that hematological and hemorheological markers differ between the sexes, and previous studies suggest sex-specific adaptations to exercise (Grau et al., 2018; Ansdell et al., 2020; Rascon et al., 2020). Nevertheless, the female participants included in this study were in the same performance category with similar finishing times and did not noticeably influence the overall results, making it reasonable to present the mixed-sex data. Future research should aim to investigate ultramarathon-induced changes separately by sex. RBC and markers of the oxidative system exhibit circadian fluctuations (O'Neill and Reddy, 2011; Budkowska et al., 2022). However, these were controlled for pre-race measurements. Analyses of the influence of sampling time (time of day of post-race blood collection) on the measured variables revealed no significant associations. This is most likely because factors such as nighttime activity, running, food intake during the night, and other influences disrupted regular circadian rhythms in this context (Kim et al., 2023; Meléndez-Fernández et al., 2023). Yet, research should consider the circadian variation of the parameters being measured.

## 5 Conclusion

This study investigated whether ultramarathon competitions affect RBC deformability and the NO-related pathway underlying these changes. The results demonstrate that RBC deformability and associated NO parameters were significantly altered by the ultramarathon, indicating sufficient mechanical stress to activate the RBC-NOS/NO pathway. Despite a reduction in haptoglobin, other hemolysis markers remained unchanged, suggesting that RBC were not substantially damaged. Furthermore, RBC oxidative stress parameters indicate that free radicals did not exert deleterious effects on RBC. Notably, although overall body hydration appeared adequate, cellular hydration decreased post-race, highlighting the importance of targeted hydration and electrolyte strategies, particularly under heat stress conditions (Knechtle and Nikolaidis, 2018; Costa et al., 2013; Cheuvront et al., 2007).

From a physiological and practical perspective, increased RBC deformability likely enhances passage through the microcirculation, improving tissue oxygen delivery and muscle perfusion. To evaluate the post-run effects of this ultra-long run on RBC, additional follow-up measurements should be performed to assess potential long-term consequences. This may be combined with improved strategies that preserve RBC health, optimize hydration, and support antioxidative defenses in ultramarathon athletes.

## Data Availability

The raw data supporting the conclusions of this article will be made available by the authors, without undue reservation.
